# Antibiotic resistance during and beyond COVID-19

**DOI:** 10.1093/jacamr/dlab052

**Published:** 2021-06-15

**Authors:** David M Livermore

**Affiliations:** Norwich Medical School, University of East Anglia, Norwich, Norfolk NR4 7TJ, UK

## Abstract

Antibiotics underpin the ‘modern medicine’ that has increased life expectancy, leading to societies with sizeable vulnerable elderly populations who have suffered disproportionately during the current COVID-19 pandemic. Governments have responded by shuttering economies, limiting social interactions and refocusing healthcare. There are implications for antibiotic resistance both during and after these events. During spring 2020, COVID-19-stressed ICUs relaxed stewardship, perhaps promoting resistance. Counterpoised to this, more citizens died at home and total hospital antibiotic use declined, reducing selection pressure. Restricted travel and social distancing *potentially* reduced community import and transmission of resistant bacteria, though hard data are lacking. The future depends on the vaccines now being deployed. Unequivocal vaccine success should allow a swift return to normality. Vaccine failure followed by extended and successful non-pharmaceutical suppression may lead to the same point, but only after some delay, and with indefinite travel restrictions; sustainability is doubtful. Alternatively, failure of vaccines and control measures may prompt acceptance that we must live with the virus, as in the prolonged 1889–94 ‘influenza’ (or coronavirus OC43) pandemic. Vaccine failure scenarios, particularly those accepting ‘learning to live with the virus’, favour increased outpatient management of non-COVID-19 infections using oral and long *t*_½_ antibiotics. Ultimately, all models—except those envisaging societal collapse—suggest that COVID-19 will be controlled and that hospitals will revert to pre-2020 patterns with a large backlog of non-COVID-19 patients awaiting treatment. Clearing this will increase workloads, stresses, nosocomial infections, antibiotic use and resistance. New antibiotics, including cefiderocol, are part of the answer.

## Introduction

The modern medical era began around 1937–42, as systemic sulphonamides and penicillin mitigated the hazard of bacterial infection, opening medical and surgical possibilities that were previously unthinkable.

**Figure 1.**
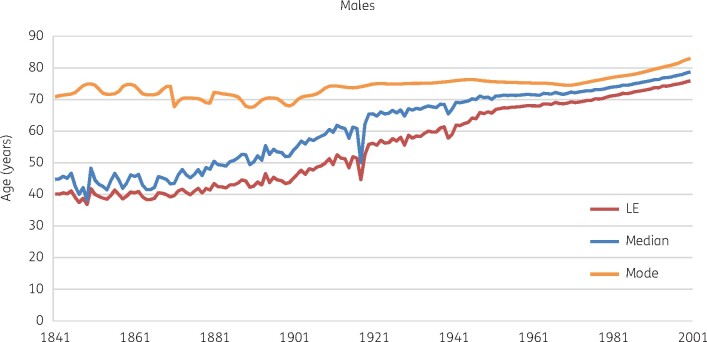
Three measures of changing lifespan for men in the UK. Data Source: Office for National Statistics.^1^ LE, life expectancy. Patterns for women are similar though life expectancy is slightly longer.

**Figure 2.**
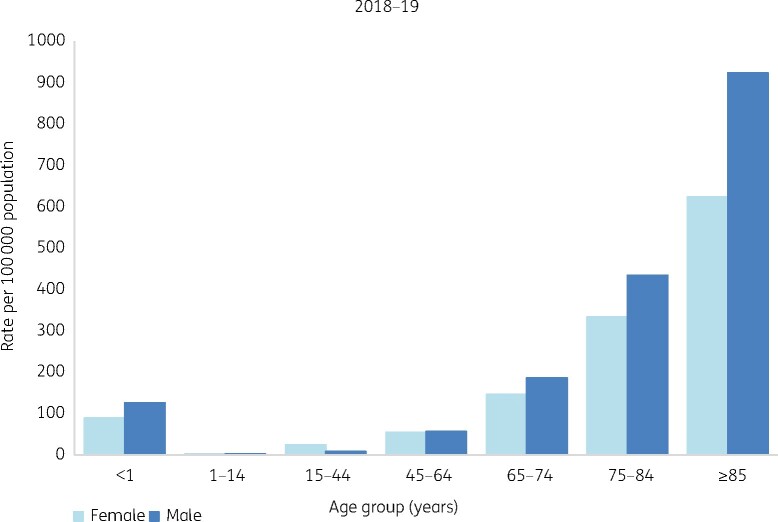
Incidence of *E. coli* bacteraemia in England and Wales, by age. Data source: PHE.^4^

Antibiotics remain the bedrock of what followed. Complex surgery, intensive care, transplants and immunosuppressive treatments all would be impossible if infection could not reliably be controlled. In the community, pneumococcal pneumonia still kills the debilitated, but no longer threatens the likes of Jane Austen’s Marianne Dashwood. Along with earlier improvements in public health, modern medicine has made early non-violent death rare in advanced societies. Mean, median and modal life expectancies have converged (Figure 1) then extended.^1^ The caveat is that late-life years of ill health have extended too,^2^ giving a growing frail elderly population with chronic illness and cognitive decline, particularly in Europe, North America and East Asia.^3^ These citizens are the frequent victims of opportunist Gram-negative bacteria, with accumulating resistance (Figure 2).^4^

Until 2020 this medical edifice grew without major viral challenge. Influenza pandemics in 1958–59 and 1968–69 killed many but were terminated by a mixture of strain ‘burnout’ and vaccination.^5^ HIV took a grim toll but was largely avoidable by personal precautions and now is medically manageable. SARS-CoV-2 has changed the dynamic, whether temporarily or more permanently.

## A brief history of COVID-19

First reports of COVID-19 seeped from Wuhan late in 2019, with the causative coronavirus SARS-CoV-2 putatively having jumped from bats in a ‘seafood’ market. Laboratory escape is plausible too, as Wuhan hosts centres for coronavirus research, but is hotly disputed.^6^

During January to February 2020, outbreaks occurred in China. By February/March infection was spreading in Iran, then Europe. The USA was hit next, with major outbreaks in the northeast, particularly New York and New Jersey. Extensive spread followed in the southern USA, Latin America and India. The pandemic peaked in northern Europe and the north-eastern USA in the early spring, with subsequent declines in infections, hospitalizations and deaths through the late spring and summer before a resurgence in the northern autumn and winter. Argentina, with the seasons reversed, showed the converse pattern, with peak deaths in October, at the end of the southern winter. With some exceptions, including a current (January 2021) upsurge in South Africa, these patterns broadly support the view that SARS-CoV-2 is transitioning from being a ‘new pandemic virus’ to an ‘endemic winter respiratory virus’, joining the four long-established coronaviruses (229E, OC43, NL63 and HKU1) that account for 10%–20% of common colds.^7^ A few countries, notably Taiwan, Australia and New Zealand, have effectively isolated themselves from the pandemic by a combination of entry restrictions and strict containment efforts whenever small clusters have been detected. Central Africa has been little affected.

Like other single-stranded RNA viruses, SARS-CoV-2 is highly mutable, with over 20 000 sequence variants described. There is current concern about particular variants, including types that first circulated extensively in the UK (VUI202012/01 or B1.1.1.7), South Africa (1.351) and Brazil (P1). These appear to spread more efficiently and, in some cases, may have modifed vaccine-relevant epitopes (see below); there are no substantiated data to indicate that they are more lethal.^8^

Most COVID-19 infection is mild, inconsequential, and self-limiting. Many only learn that they have been infected when they are found seropositive. Even when virus is detected by RT-PCR, half record no symptoms.^9^ Among those who do develop symptoms—predominantly fever, cough and shortness of breath along with loss of taste and smell—recovery generally follows after 1 week. But, for a minority, pulmonary symptoms worsen, necessitating hospitalization and, in extremis, supplementary oxygen or ventilation.^10^^,^^11^ Death occurs in 40%–50% of ICU cases,^12^ increasing with age, male gender, obesity, dementia, diabetes and cardiovascular or pulmonary disease.^13^

Estimation of fatality rates is fraught, since most mild infections pass unrecorded. In October 2020, the WHO suggested that c. 10% of the world’s population had been infected,^14^ and that deaths had then reached 1 million. This indicated an infection fatality rate of around 0.13%. Ioannidis,^15^ using seroprevalence data as the denominator, estimated 0.15%–0.2%. These statistics are reassuring but carry four caveats: (i) the proportion is significantly higher in countries with a large elderly population; (ii) sufficient severe cases can arise to overwhelm local or national ICU capacity, again especially if there is a large vulnerable elderly population;^16^ (iii) outbreaks in elderly care facilities can kill extensively, as in the UK, Sweden, New York, Italy and Spain;^17–19^ and (iv) even low mortality rates translate to numerous deaths in large populations. The aspects have dominated political debate, media coverage and policy response. As of this writing (January 2021) the UK NHS has around one-third of its beds occupied by patients infected with SARS-CoV-2, including more than half of its ICU beds, and is clearly showing stresses, emphasized in rolling 24 h news bulletins. Cold review of actual numbers gives a different perspective. From a UK population of 67 million, roughly 1.1 million (2%) were estimated to be infected with SARS-CoV-2 in early January,^20^ and just 3000—1 citizen in 22 000—were sufficiently sick to need ICU care. In other words, the central issue is a shortage of ICU beds for the minority who become severely ill, and staff to support them, not that COVID-19 has a high fatality rate.

**Figure 3.**
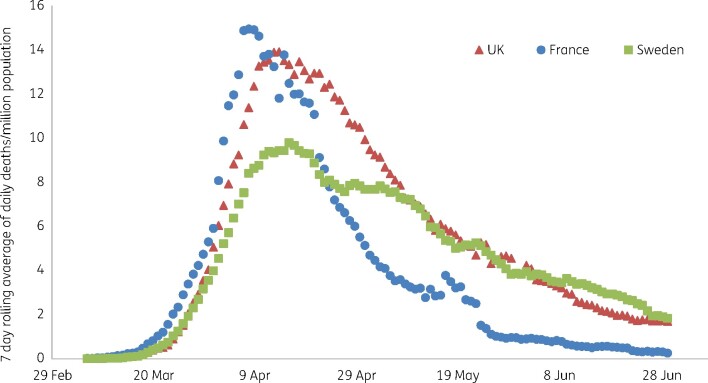
First wave deaths from COVID-19 in France (strict lockdown; 13.8% Q2 fall in GDP), UK (moderate lockdown; 20.4% Q2 fall in GDP) and Sweden (no lockdown; 8.6% Q2 fall in GDP).^96^

Most governments across Europe, North America and South America have enacted repeated ‘lockdowns’, closing the economy, confining populations and mandating social distancing. Reductions in deaths are attributed to these actions in China (strict lockdown), Europe and New York (varying strictness).^21^ There is, however, considerable scope for scepticism. In the initial spring wave, UK deaths peaked on 8 April,^22^ whereas lockdown began on 23 March, suggesting that new infections were already declining, assuming ≥19 days from infection to death (5–6 days incubation, >8 to hospitalization, ≥6 to death). Moreover, there is a remarkable similarity between the spring trajectories of death rates per million population between France, with a strict lockdown, the UK, with a less severe lockdown and Sweden, which had no lockdown beyond general advice on social distancing and restrictions on large events and bar-counter service (Figure 3). The likely explanation is that viral seasonality, not lockdown, underpinned the declines in each country. In an extensive analysis, De Larochelambert *et al.*^23^ reviewed deaths against lockdown stringency for 160 countries, finding little relationship and concluding that death rates largely reflected whether a country was in the temperate zone, typically had few deaths due to communicable diseases, and had a large elderly population for whom life expectancy was no longer extending. Strict lockdowns in seven Danish counties, enacted following discovery of a new variant in mink, had no greater effect than milder restrictions in four adjacent counties;^24^ death and infection trajectories in North and South Dakota are almost superimposable, despite more extensive business closure restrictions (and mask mandates) in the former. Lockdowns have only worked convincingly where they were enforced very strictly against outbreaks that were tiny in global terms, as in Melbourne, or where, as in China, they approximated to classical quarantine, by extracting and confining those found infected.

## Immediate impacts on antibiotic use and resistance

Most non-hospitalized COVID-19 patients receive no antibiotics. On the other hand, antibiotics—typically those used for community-acquired pneumonia (i.e. amoxicillin/clavulanate + macrolide; ceftriaxone + macrolide or levofloxacin)—are prescribed to hospitalized cases, though few have evidence of bacterial infection.^25^ Thus, Rawson *et al.*^26^ estimated that 72% of hospitalized COVID-19 patients received antibiotics but only 8% had bacterial infection and Langford *et al.*^27^ published similar figures. This suggests poor stewardship. Others note that bacterial coinfection is rarer than in influenza^28^ whilst a Swiss study found that ‘early’ antibiotics, before ICU transfer, had little benefit.^29^ Some hospitals initially administered hydroxychloroquine plus azithromycin against COVID-19 itself, though any benefits, and their mechanism, are disputed and the therapy has fallen into disfavour.^30^^,^^31^

ICU COVID-19 patients are usually intubated and face the risk of ventilator-associated pneumonia, mostly involving the Enterobacterales, *Staphylococcus aureus* and non-fermenters typical of this infection. Across five UK ICUs we found *Klebsiella pneumoniae* and *Klebsiella aerogenes* unusually prevalent in COVID-19 patients,^32^ whereas a single-hospital French study found an excess of non-fermenters.^33^

Ventilated COVID-19 patients often receive multiple antibiotic courses. At the height of the pandemic, stewardship policies were overridden,^26^ with ICU capacity increased. A Spanish hospital reported increased antibiotic use.^34^ Such data lead to concern that resistance may proliferate in hospitals as a result of COVID-19 pressures, though with scant evidence that it has actually done so. Resistance drivers in the community potentially may increase too. More general practice consultations are remote, and pre-COVID-19 studies suggest that US community physicians are more willing to prescribe antibiotics when consulted online for children^35^^,^^36^ though not for adults.^37^ Delivery of childhood vaccines has been disrupted,^38^ favouring resurgence of multiresistant vaccine serotypes of *Streptococcus pneumoniae*. Disruption of TB treatments will promote recrudescence, resistance and transmission of resistant variants, potentially leading to future treatment difficulties. This is an issue e.g. in India, where TB kills over 420 000 p.a., or around 2.5-fold more than COVID-19 to date (January 2021).^39^ Dentists—long discouraged from antibiotic use—were reduced to the options of antibiotics, analgesics and extraction, with aerosol-generating procedures forbidden.^40^^,^^41^

However, countervailing forces apply, reducing pressure for resistance. First, much non-COVID-19 hospital activity ceased during peaks of COVID-19 activity.^42^ In some jurisdictions, particularly the USA, hospital staff were laid off.^43^ The complex patients who are most vulnerable to multiresistant Gram-negative bacteria were no longer hospitalized. In the UK more people died at home and in care homes rather than in hospitals, where they likely would have received antibiotics.^22^ IV antibiotic use in English hospitals, measured as DDDs, was 32% lower in April–May 2020 than in April–May 2019 (P. Howard, Leeds Teaching Hospitals NHS Trust, personal communication). Similarly, wholesale IV antibiotic shipments to US hospitals, as DDDs, declined 30.7% in the same comparison (A. Carr, Needham & Company LLC, personal communication) with only 4/36 products showing increases. These data suggest reduced use, though we cannot exclude distortions from stock management inside hospitals, and the decline was only 6.9% if the month of March was added to the comparisons. A more recent report, comparing January to November 2020 with January to November 2019, indicates reduction in unit sales of systemic antibiotics as follows: Spain, 2.1%; France, 3.6%; Germany, 9.3%; Italy, 14%; and the UK, 14.5%.^44^ Reports of *Escherichia coli* bacteraemia to England’s mandatory surveillance in the July to September quarter of 2020 were 13.4% below those of the same quarter of 2019, sharply reversing a rising trend.^45^ The likely explanation is that many septic patients, who ordinarily would present to A&E, are failing to do so and are failing to receive IV antibiotic therapy. They may be represented among the persistently increased numbers of citizens presently dying at home rather than in hospitals.^46^ Changes in incidence are much less marked for bacteraemias involving pathogens that are mostly healthcare acquired, specifically *K. pneumoniae* and *Pseudomonas aeruginosa*.

Second, ICU triage, as applied at the height of the pandemic,^47^^,^^48^ militated against the ‘frequent flyer’ patients likely to be pre-colonized with multiresistant opportunists, favouring hospital-naive patients more likely to retain a susceptible flora.

Third, international travel has been dramatically curtailed, and this must reduce the transfer of resistance. London private hospitals ordinarily admit patients from the Middle East, frequently already colonized with resistant Gram-negative opportunists.^49^ This has stopped. Travellers e.g. to India commonly become colonized by ESBL-producing *E. coli*.^50^^,^^51^ Again, such travel has essentially ceased. Social distancing and travel restrictions reduce opportunities to catch and import ‘super gonorrhoea’;^52^^,^^53^ though closure of genitourinary medicine clinics^54^ will facilitate the spread of any already circulating, and a study in Milan indicated no reduction in presentations for acute syphilis and gonorrhoea in early 2020 compared with 2019.^55^

Social distancing and masks may impact community transmission of respiratory infections, reducing demand for antibiotics. The elderly often acquire pneumococci from grandchildren^56^ and will not do so if families cannot meet. In Italy, discontinued medical monitoring of otitis media-prone children led to reduced antimicrobial prescriptions in the late winter, without apparent harm.^57^

A final aspect, of uncertain impact, is the COVID-19-directed use of personal protective equipment. This might be expected to diminish cross-infection, but the inconvenience of changing between patients increased MRSA transmission in the 2003 SARS outbreaks in Canada and Singapore.^58^^,^^59^

## What next? Possible scenarios

There are several plausible futures. These are set out below and their implications for resistance, summarized in Table 1, are then considered. There also are extreme possibilities, outlined briefly in the concluding paragraphs of this paper.

**Table 1.** Implications of different scenarios for resistance

Scenario	Central prediction on COVID-19	Sustainable	Push towards more treatment in the community with oral, OPAT and long *t*_½_ agents	Surge of hospital activity to clear backlog	Travel; import of resistance
Vaccine overwhelmingly successful, and perceived as such	Burden no greater than seasonal influenza with this politically acceptable	Yes	Brief: until population vaccinated 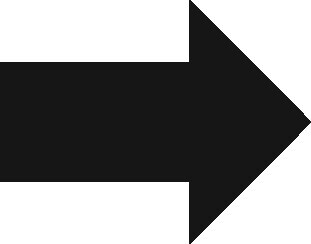	Early 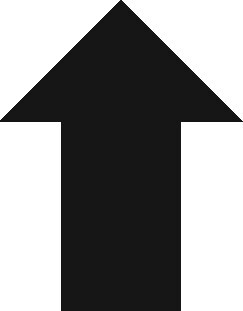	Briefly reduced, then normalized 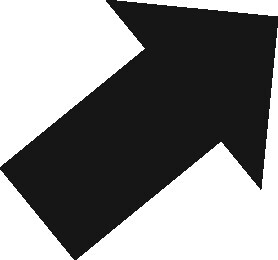
Vaccine failure or perceived failure. Prolonged emphasis on track and trace	Control requires eternal vigilance but is achieved and maintained	Doubtful	Brief (if successful): until COVID-19 reduced to low incidence 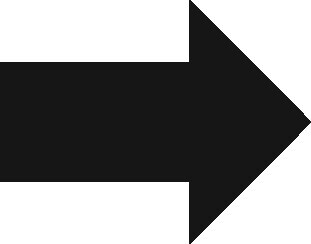	Early (if suppression successful) 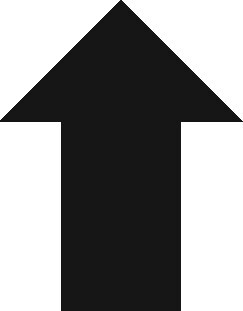	Reduced for prolonged period 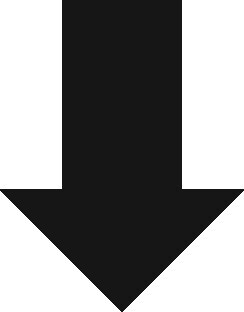
Vaccine failure. Acceptance that virus is established, endemic and that lockdowns are ineffective or cause unacceptable collateral damage	Successive COVID-19 waves, ending in herd immunity; significant further direct mortality	Yes	Extended: until population immunity dominates 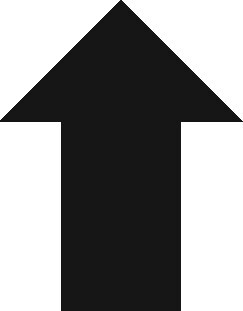	Delayed 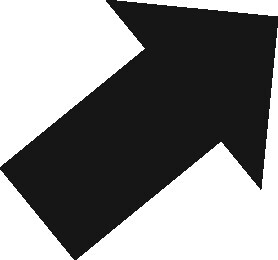	Steady reversion to normality 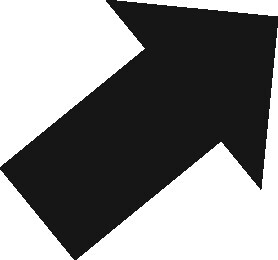

Arrows indicate predicted change in selection pressure from the pre-COVID-19 situation: upward, increased selection pressure; horizontal, reversion to status quo ante; downward, reduced selection pressure.

Vaccines directed against SARS-CoV-2 (Table 2) have been developed at impressive speed. Based on interim analyses of ongoing trials, several have been given emergency use authorizations in multiple jurisdictions. Those deployed in Europe and North America are ‘new-technology’ mRNA and adenovirus vector products targeting the SARS-CoV-2 spike protein, which is crucial to viral receptor binding. Classical inactivated virus vaccines have been developed in China and are finding use in South East Asia, Latin America and the Middle East. Deployment is most advanced in Israel, with most (>80%) of the population now vaccinated using the Pfizer BioNTech product, but is progressing rapidly e.g. in the UK, UAE, USA and Chile.

**Table 2.** Vaccines against SARS-CoV-2

Vaccine	Manufacturer	Type	Efficacy	Notes	Reference
BNT162b2	Pfizer BioNTech	mRNA	95%		^122^
mRNA-1273	Moderna	mRNA	94.1%		^123^
Sputnik	Gamaleya Institute	adenovirus vector	91.4%		^124^
ChAdOx1 nCoV-19	AstraZeneca/Oxford University	adenovirus vector	53.4%–90.0%	efficacy varied with subgroup, dosage and dosage interval	^125^
BBIBP-CorV	Sinopharm	inactivated virus	79%–86%		^126^
CoronaVac	Sinovac	inactivated virus	50.4%		^127^

Although early results are promising, considerable uncertainty remains. First, since use is based on interim trial analyses the duration of protection is unknown. Post-infection immune responses to the classical coronaviruses (229E, HKU1, NL63 and OC43) fade swiftly, restoring vulnerability to infection, though this is generally mild.^60^ Infection-induced IgG to SARS-CoV-2 declines rapidly too,^61^^,^^62^ especially in asymptomatic cases, suggesting a similar risk, though clinically manifest reinfections seem rare, perhaps owing to persistent T cell-mediated immunity.^63^ Secondly, there is uncertainty about vaccine responses in the vulnerable elderly with ‘adaptive immunosenescence’.^64^ Thirdly, it is uncertain whether the vaccines will prevent infection or will reduce severity whilst leaving infected vaccinees as vectors of infection. Last, some emerging virus variants have mutations affecting the spike protein, and it is uncertain whether the present vaccines will reliably cover all present and future variants.^8^

The optimistic scenario is that vaccines overwhelmingly succeed, reducing the threat of SARS-CoV-2 at least to that of seasonal influenza (which typically has 10 000–30 000 attributed deaths annually in England),^65^ and that the public accept this situation, allowing a return to normality. At worst, in this scenario, an annual booster shot will be needed, particularly for the elderly and those caring for them, and perhaps with some adaptation to cover prevalent variants, as with influenza vaccines.

The pessimistic scenario is that vaccines provide only modest and brief protection, most probably owing to the proliferation of diverse spike protein variants and/or to general failure to protect the most vulnerable elderly. Failure might also arise if the public, after a year of saturation propaganda, can be satisfied by nothing less than ‘zero COVID’.

Substantial vaccine failure (or unrealistic demands for complete suppression) could be met with indefinite restrictions on social interactions along with extensive track and trace systems. Incoming travellers, including returning nationals, would require testing or quarantine; outgoing travellers would enter a dangerous world unless all countries follow this approach (which they are not doing). The strategy may be sustainable for a remote island, possibly New Zealand, but seems unfeasible in the long term for a trading nation, let alone for a continental union with free movement and varied national approaches to COVID-19.

The alternative response to vaccine failure is to accept that SARS-CoV-2 has become endemic and must circulate, potentially in the form of diversifying spike protein variants that facilitate reinfection. Repeated exposure, together with modestly protective vaccines, should progressively reduce disease severity, especially among the young, who would age with SARS-CoV-2 as we all do with the four long-established coronaviruses. The difficulties with this model are (i) how best to protect the present cohort of most-vulnerable elderly, who lack both prior exposure and the ability to adapt, and (ii) how to re-educate a public that has been ‘trained’ to believe COVID-19 to be far more lethal than is actually the case.^66^

There is one tantalizing hint of how a future that accepted spread might unfold: the 1889–94 ‘Russian influenza’ pandemic. This is conventionally attributed to H2N2 or H3N8 influenza A,^67^^,^^68^ based on the serology of elderly patients tested decades later. An alternative hypothesis is that coronavirus OC43 was responsible, having evolved apart from a bovine coronavirus shortly beforehand.^69^ Like COVID-19 and unlike influenza, the 1889–94 infection selectively killed men, spared children^70^ and caused loss of taste and smell.^71^ Unlike earlier influenza epidemics it gave repeating similarly sized waves over 5 years, a point thought unusual at the time, and which seems exceptional compared with any influenza epidemic in the preceding 200 years or the subsequent 130.^72^^,^^73^ Such a prolonged pandemic fits a model whereby prior exposure to other coronaviruses gives partial cross-protection, as now postulated for SARS-CoV-2,^74^^,^^75^ but with cohorts regaining vulnerability as immune responses diminished, and perhaps experiencing more than one OC43 infection as immune-escaping mutants were selected. This is speculation, but the parallels are intriguing.

*If* correct and *if* predictive (two big ‘ifs’!), it implies that coevolution of man and virus may take half a decade to achieve equilibrium. Even today OC43 can cause lethal care home outbreaks.^76^

## Implications of the scenarios for antibiotic usage and resistance

### 1. Vaccine success

If vaccines prove overwhelmingly successful there should be a progressive and increasingly exuberant return to the ‘old normal’ in human behaviour and (assuming solvency) travel. Hospitals will face a backlog of elective procedures, along with patients who, fearful of nosocomial COVID-19, had postponed seeking healthcare; one analysis suggests that this backlog may amount to almost 5 million hospital treatment episodes in the UK alone.^77^ Some will have more severe disease, including more advanced cancers, than would ordinarily be the case. Unless additional hospitals can be commissioned, and (the greater challenge) staffed, there will be considerable workload pressures, which are correlates of increased nosocomial infections,^78^ antibiotic use, and resistance. In short, once healthcare and travel revert to full capacity, more resistance should be expected.

A partial counterpoise will be the numbers of previously heavy users of healthcare who succumbed to COVID-19 or (because they could not access treatment in the COVID-19-dominated period) to other illnesses. UK excess mortality from March to June 2020 was 30% above normal, with half the deaths falling among care home residents.^79^ Their demise will reduce hospital demand, but this factor will be small: the great majority of the highly vulnerable population have survived the pandemic. 

### 2. Perceived vaccine ‘failure’: long-term track and trace seeking ‘zero COVID’

The aim here, following vaccine disappointments, would be to suppress COVID-19 sufficiently that normality of a sort resumes within a closed system, as presently in Taiwan, Australia or New Zealand, all of which achieved early control of viral spread meaning that their hospitals are not under the pressures seen elsewhere. If successful, the medium-term implications for hospital antibiotic utilization would resemble the vaccine case. In the short term, the pressures would be rather different and would continue to resemble those that have pertained in the pandemic itself, both in respect of hospital workload being dominated by COVID-19 and with reduced hospital capacity caused by the needs (i) to socially distance beds, (ii) to cohort patients according to COVID-19 status, and (iii) for numerous staff to self-isolate following track and trace alerts. These factors may drive a shift to outpatient antibiotic therapy and long dosage-interval antibiotics, followed by rise in use, selection pressure and bacterial cross-infection once COVID-19 comes under control and hospitals move to clear their backlog. Such a model must assume drastic long-term reductions in international travel, as it would not be feasible to allow free movement to and from countries lacking similarly stringency. This would impede the transnational flow of resistant bacteria.

The issues with this model are not its implications for antibiotic resistance, which are broadly positive, at least in the short term, but its feasibility and its sustainability. Track and trace systems have, so far, only worked in countries where COVID-19 gained little initial traction, not those, such as the UK, USA and the EU states, where the virus has become endemic and prevalent. In these latter polities, track and trace has been overwhelmed or confounded by undetected cases, spurious late positivity in recovered patients,^80^ poor concordance between repeat tests^81^ and poor agreement between different types of test.^82^ Once infection rates are low, false positives are apt to outnumber true positives, even for a test with e.g. 99% specificity, reducing the positive predictive value.^83^ The failure of track and trace is illustrated by the extent to which governments have resorted to repeated lockdowns that they had sworn, after Spring 2020, to eschew.

In the view of this author, vaccines would have to come close to being successful, greatly reducing disease prevalence, before the approach becomes practicable. And, if these conditions pertain, it becomes disproportionate to prioritize COVID-19 compared with other infections, notably influenza, that remain significant causes of death in the same demographic. What is more, the economic and social costs will mount as other countries, eschewing this approach, abandon restrictions and their contingent costs. Closed defensive economies rarely prosper. These issues, albeit without the issues of healthcare backlog, will have to be faced also by those countries that have been most successful at suppressing COVID-19 during 2020. Should they deploy a suboptimal vaccine, accepting that they will then have COVID-19 and COVID-19 deaths, or should they remain closed?

### 3. Vaccine ‘failure’: community control relaxed or abandoned

Given the massive ‘sunk cost,’ control abandonment is now likely only after multiple vaccine disappointments and as the social and economic cost of lockdowns becomes obvious and painful, even to those who presently believe in their efficacy and virtue.

Further viral waves would then be anticipated, largest in countries that initially suppressed COVID-19 most effectively or, more randomly, in those where immunologically distinct variants emerge. If the 1889–94 ‘influenza’ is a model, spikes of infection might extend over years. Vaccines, whilst failing to prevent COVID-19, may mitigate severity and treatments will likely improve. Dexamethasone reduces mortality^84^ in severely ill patients, and inhaled interferon-β may reduce progression to severe disease.^85^ Clinical manageability may encourage governments to reduce suppression.

Even so, hospitals will still be hazardous, or be *perceived* as hazardous, extending pressure to use oral outpatient parenteral antibiotic therapy (OPAT) and long *t*_½_ antibiotics. Since this period will be longer than under other scenarios, there will be more impetus to develop such therapies. Single-dose IV oritavancin and dalbavancin give near-universal antistaphylococcal coverage, as do (multidose) oral oxazolidinones, delafloxacin and omadacycline.^86^ Oral cephalosporin/β-lactamase inhibitor combinations and (carba)penems—sulopenem and tebipenem—are in development,^87^^,^^88^ targeting ESBL producers. Although sulopenem disappointed in complicated urinary tract infections (cUTI),^89^ it proved effective in uncomplicated urinary tract infections,^90^ whilst tebipenem was found to be as effective as ertapenem in cUTI.^91^ Of particular note are combinations of ceftibuten with the oral boronate QPX7728, which inhibits serine and metallo carbapenemase (except IMP types) as well as ESBLs and AmpC enzymes.^92^

Gradually, normality will return. And maybe sooner than the 1889–94 analogy suggests, given the boost that even partially effective vaccines may provide. Public fear will subside as the huge excess of mild infection is better appreciated. Hospitals, society and travel will revert to pre-pandemic patterns though after a disruption that may persist for several years.

Ultimately all these models predict that COVID-19 will, more or less quickly, decline in importance. As it does so, old concerns will re-emerge, mirroring Churchill’s^93^ observation after WW1:

*‘The position of countries has been violently altered. The modes of thought of men, the whole outlook on affairs, the grouping of parties, all have encountered violent and tremendous change… But as the deluge subsides and the waters fall short, we see the dreary steeples of Fermanagh and Tyrone emerging… The integrity of their quarrel is one of the few institutions unaltered in the cataclysm…’*

**Figure 4.**
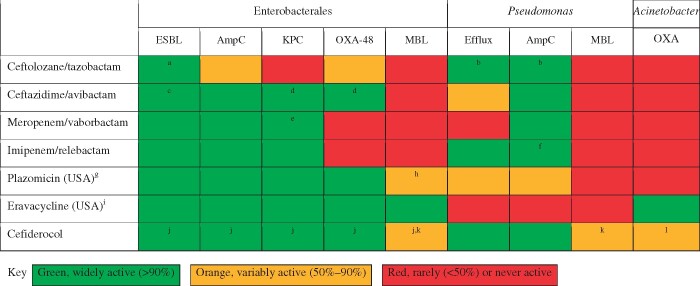
Activity of recently licensed (USA and EU/UK) agents against problem groups of Gram-negative bacteria. Green, widely active (>90%); orange, variably active (50%–90%); red, rarely (<50%) or never active. ^a^Trial evidence of efficacy.^128^^b^In-use evidence of clinical activity against *P. aeruginosa* likely, based on phenotypes, to have these mechanisms.^129^^c^Trial evidence of efficacy.^130^^d^In-use evidence of efficacy and of better outcomes than colistin combinations.^131^^,^^132^^e^Trial evidence of better outcomes than colistin combinations.^133^^f^Trial evidence of activity against imipenem-resistant *P. aeruginosa*, likely to have owed their phenotypes to combination of loss of porin OprD and expression of AmpC.^134^^g^Licensing application withdrawn in EU. ^h^Many isolates with NDM carbapenemases co-produce ArmA or RmtB 16S rRNA methyltransferases, conferring broad aminoglycoside resistance including plazomicin.^135^^i^Good *in vitro* activity against carbapenemase-producing Enterobacterales, but trial failures in cUTI.^136^^j^Trial evidence of activity.^137^^k^MICs raised for isolates with NDM carbapenemase compared with those for isolates with other carbapenemases; the proportion of these that count as resistant will depend on the breakpoints used.^138^^l^*In vitro* activity, but excess mortality in CREDIBLE-CR study compared with colistin combinations, associated with *Acinetobacter baumannii,* suggesting the need for caution.^139^

And, in the present context, multiresistant Gram-negatives will renew their challenge. Those seeking a review of prevalent types are directed to the article by Bush and Bradford,^94^ those wishing to appreciate differing threats of ‘carbapenem resistant’ and ‘carbapenemase-producing’, to our own publication.^95^ Figure 4 of the present paper summarizes the activity of recently licensed agents against important resistance types, noting where there is demonstrated clinical evidence of efficacy.

## Conclusions

COVID-19 is not a great historical pandemic. During 2020 it was reportedly involved in around 1.8 million (3%) of the 60 million deaths that occurred worldwide, and the world population *rose* by 80 million.^96^ The 1347–50 Black Death, for comparison, reduced the European population by 33%–60%, with recovery taking 150 years. On 29 September 1918, the troopship SS *Leviathan* cleared New York with 11 800 aboard. When she docked at Brest 10 days later, 2000 were sick with influenza, 1000 were stretchered ashore and 80 were dead; 15 more died in France.^97^ For comparison, a COVID-19 outbreak on the USS *Theodore Roosevelt* infected at least 1200 from a complement of 4000.^98^ One died. The 1889–94 pandemic killed 125 000 in the UK, 27 000 in its 1889–90 wave. This was from a population of 33 million, or around half of today. Some social scientists blame the influenza for *fin de siècle* angst,^99^^,^^100^ but life continued. Gilbert and Sullivan’s * Gondoliers* opened on 7 December 1889, days before the first case, playing continuously until April 1891. Prince Eddy—second in line to the throne—succumbed on 14 January 1892; *Lady Windermere’s Fan* opened in the February. In October 1918, the Allies’ ‘100 Days Campaign’ crept bloodily eastwards, defeating the German army just as the pandemic peaked.^101^^,^^102^ Across the lines, Berlin alone recorded 1700 influenza deaths on 18 October,^103^ but retained sufficient energy for street revolution to erupt in November.^104^ Our forebears, lacking virology, would have mistaken 2020 for a ‘bad flu year’, mourned their dead, but carried on.

Where COVID-19 is unique is in hitting a modern medicalized population with many elderly and vulnerable, and in humanity’s reaction. Never before was it policy to shutter the economy or to confine the healthy. The WHO’s pandemic influenza plan of 2019 makes no mention of lockdown as a strategy^105^ and the approach was expressly dismissed in the 1957 influenza pandemic.^106^^,^^107^

It will be for future historians to assess the wisdom or folly of the policies adopted in 2020–21, but it is already arguable that our response generated more harm than the epidemic, leading to impoverishment, delayed treatment and increased mortality for other (e.g. cardiovascular) conditions, disrupted educations and mental illness.^108–111^ A particularly extensive review of the harms of lockdown is provided by Joffe.^112^ Many ‘saved’ by lockdowns had little time to live: someone *entering* a care home in the UK ‘expects’ c. 30 months, and care home residents account for half the UK deaths.^113^ Those whose prospects are blighted by the response to COVID-19 span the age spectrum. Unless vaccination is successful, or societies are prepared to accept indefinite and stultifying restrictions on liberty, the epidemic must ultimately run its course.

Against this ‘big picture’, effects on antibiotic resistance are a sideshow. Sharp reductions in COVID-19-unrelated medicine, IV antibiotic use and travel are *reducing* short-term selection pressure nationally, though selection may be locally increased in stressed ICUs. The longer-term effects depend on the success of vaccines or, if they fail, on our response to this failure. If vaccines succeed overwhelmingly, a hectic period will follow as hospitals address a backlog, with some patients sicker than had they been treated earlier. Resulting pressures will promote resistance. If vaccines fail, or if unrealistic hopes lead to a perception of failure, a more atomized society will persist. This will favour oral, OPAT and long *t*_½_ antibiotics, reducing hospital-centred selection and cross-infection. Travel will be reduced, limiting import of resistance. But such an approach is unsustainable except in an island choosing indefinite isolation. The dénouement, sooner or later, will be relaxation, further COVID-19 waves, perhaps by vaccine-evading variants, then recovery and normalization.

Some shifts seem set to be maintained, notably more homeworking, which may reduce circulation of other respiratory infections and the contingent, often unwarranted, community demand for antibiotics. In hospitals, all ‘likely’ scenarios favour a short-term reduction in resistance selection, then a bounceback. Ultimately, old challenges will renew, including with carbapenemase producers. Newer antibiotics, including cefiderocol, address these.

Last, there are extreme futures, where economic damage arising from lockdowns or failure of the ‘modern monetary theory’ used to finance COVID-19 responses precipitates civil unrest, loss of confidence and a flight to gold. Lebanon—already in political turmoil in 2019—exemplifies COVID-19 tipping a precarious situation over the edge. During 2020 the lira fell 85% on the dollar, inflation hit 50% *monthly* and the government was unable to pay healthcare providers. Hospitals suffered blackouts. An early ‘total shutdown’ was followed by an accelerating case tally^114^^,^^115^ and a further shutdown, though it was hard to see how this could be financed, or a good outcome achieved, even without the devastating explosion of 4 August.^116^ Experience in Libya and Syria shows that carbapenemase-producing bacteria can proliferate in times of chaos.^117^^,^^118^ The inability of a bankrupt Argentina to pay for antibiotics in 2003 was associated, briefly, with reduced use^119^ though also with worse outcomes for non-infectious conditions,^120^ and increased mortality in infections.^121^

If future society is to prosper and to be able to afford modern medicine, it is vital that we avoid such futures, for their human cost will greatly exceed than any toll arising from the virus itself.
